# Neuroinfection as a Mask of Lung Cancer: A Case Series

**DOI:** 10.1155/2016/6061350

**Published:** 2016-04-30

**Authors:** Beata Kuklińska, Anna Moniuszko-Malinowska, Robert Mróz, Sławomir Pancewicz, Joanna Zajkowska

**Affiliations:** ^1^Department of Lung Diseases and Tuberculosis, Medical University in Białystok, Białystok, Poland; ^2^Department of Infectious Diseases and Neuroinfections, Medical University in Białystok, Białystok, Poland

## Abstract

*Introduction*. The diagnosis of lung cancer may still be difficult due to the fact that the first symptoms very often mimic symptoms of other diseases.* Case Presentation*. In this paper we present two cases, in which initial diagnosis of neuroinfection delayed proper diagnosis.* Conclusion*. Based on our experience we concluded that neurological symptoms in the area endemic for tick-borne diseases suggesting neuroinfection require careful differential diagnosis. Moreover, neurological symptoms in heavy smokers may be associated with metastases of lung cancer.

## 1. Introduction

Constant development of medicine, diagnostic techniques, and antibiotic therapy contribute to quick diagnosis and treatment of the infections of nervous system. The diagnosis may still be difficult due to the fact that the first symptoms very often mimic symptoms of other diseases [1]. In this paper we present two cases, in which initial diagnosis of neuroinfection delayed proper diagnosis.

## 2. Case 1

61-year-old patient, smoker, with a history of specific process (in 1992) and COPD, was treated with painkillers by GP in April 2015 because of headache for 2 weeks. After a week nausea and pain in the upper abdomen appeared. He experienced numbness of the fingers and balance disturbance. He was admitted to the Department of Neuroinfections with suspicion of tick-borne encephalitis, as he was bitten by tick.

On admission patient complained of headache, vertigo, numbness of the fingers, and nausea. Physical examination revealed neck stiffness, positive Romberg test, positive Kernig symptom, impaired sensation in the left upper limb, hushed murmur of prolonged exhalation, and a sign after tick bite on the left popliteal area. Cerebrospinal fluid was xantochromic and clear, protein 162.5 mg/dL (15–60), albumin 145.6 mg/dL (10–30), and cytosis 44 cells/*μ*L (0–5), with predominance of lymphocytes: 79% (40–80).

Based on anamnesis, clinical picture, and CSF results lymphocytic meningitis was diagnosed. After two days of treatment (mannitol, painkillers) the patient's condition deteriorated with the appearance of balance disturbances.

**Figure 1:**
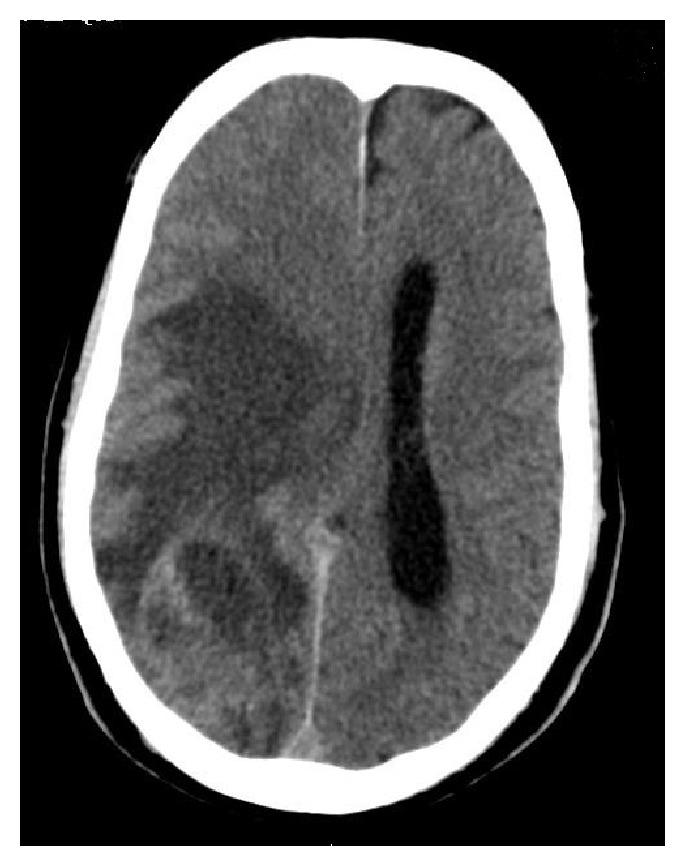
Head CT: a heterogeneous lesions, mostly marginal contrast enhancement (50 × 40 × 40 mm) with large mass effect, a vast zone of grade III edema, covering the structures of the right hemisphere and displacing the middle cerebral structures towards the left (11 mm) in the parietooccipital part of the right hemisphere of the brain. A similar minor (13 mm) change was present in the left frontal lobe with a small area of swelling and in the subtentorial area, in the left cerebellar hemisphere (16 mm).

Head CT revealed in the parietooccipital part of the right hemisphere of the brain heterogeneous lesions, mostly marginal contrast enhancement (50 × 40 × 40 mm) with large mass effect, a vast zone of grade III edema, covering the structures of the right hemisphere and displacing the middle cerebral structures towards the left (11 mm). A similar minor (13 mm) lesion was present in the left frontal lobe with a small area of swelling and in the subtentorial area, in the left cerebellar hemisphere (16 mm). These lesions suggested metastates (Figure 1).

In chest X-ray widening knobby outline of the right recess was found. Chest CT showed lesion (29 × 22 mm) in the upper lobe of the right lung. In the upper fields of both lungs there were nodulo-linear opacities with small calcifications. In segment 6 of the left lung irregular linear and patchy opacity merging with linear pleural shadings were present. There were nodular densities of 7 mm diameter in the right lung. In the mediastinum there were small lymph nodes (max. 7 mm), in the right hilum (12 × 9 mm and 11 × 6 mm).

The histopathological examination allowed the final diagnosis: small-cell lung cancer.

## 3. Case 2

Man, aged 57, farmer, smoker, and previously healthy, referred to the GP in February 2015 due to left upper limb pain, most severe in the elbow, persistent since January. He could not deny the injury (perhaps at work on the farm). Neither swelling nor redness were found. He received Profenidum 2 × 100 mg for 1 week with slight improvement. He was referred to the Department of Neurology with the diagnosis of neuropathy of the ulnar nerve. The clinical picture suggested neuroborreliosis (radicular syndrome), as the patient was the resident of endemic area for tick-borne diseases and was exposed to tick bite. During the following days numbness of fingers of the left upper limb appeared. The straightening of the upper limb at the elbow was not feasible due to the severe pain.

X-ray of elbow joints and bones of the forearm revealed osteolytic lesions. Brain CT presented cyst (28 mm × 40 mm), with inhomogeneous marginal contrast enhancement in the right occipital lobe with the vast area of hypodension in the parietooccipital region of the lobe, the area of similar nature (36 mm × 27 mm) in the right hemisphere of the cerebellum with hypodense zone of edema, metastases. Hyperdense area (11 mm × 7 mm) in the midline at the base of frontal lobes, with heterogeneous contrast enhancement suggested meningioma. Midline brain structures were displaced by 9 mm to the left side, the right lateral ventricle and the fourth ventricle were narrow, and left lateral ventricle was extended. The grooves of the right hemisphere were oppressed and had edematic characteristics. Features of intussusceptions were observed. Chest X-ray showed nodular shade in the upper right lung.

On admission to the Department of Lung Diseases and Tuberculosis patient complained of pain and weakness of left upper limb. Chest CT showed enlarged lymph nodes in the mediastinum. In the top of the right lung heterogeneous mass of irregular lumpy shape with uneven margins and calcifications linked to the upper pole of the right lung cavity was observed. The distal section of the artery was caught into the upper lobe of the right lung (tumor mass). The right main bronchus was modeled by the lymph nodes. In segment 3 in the front of the right lung there was a lumpy mass with smooth contours (26 × 19 mm), and in segment 6 parahiliar nodular lesion (14 × 13 mm) there was suspicion of metastases. In the left adrenal gland metastases were stated.

Bronchofiberoscopy showed no abnormalities. Chronic inflammation was diagnosed. Ultrasonography of the abdomen showed metastases in liver. After cytology examination the final diagnosis of low-differentiated cancer, probably squamous, was established.

## 4. Discussion

The clinical picture of lung cancer is often nonspecific. The clinical symptoms and radiographic changes in the lungs may be nonspecific. Particularly common in the literature are descriptions of cases of the advanced stage lung cancer, where the disease appears under a “mask” clinically dependent on the presence of particular groups and syndromes and their configuration. Clinical symptoms of lung cancer are heterogeneous due to the anatomical location of the primary tumor, histological weave, biological properties of the tumor stage, and the presence, number, location, and size of metastases [2].

Patients with lung cancer at an early stage often do not report any problems. Chronic cough, shortness of breath, and recurrent respiratory infections are linked to smoking. They do not consider them as pathological symptoms. Hence uncharacteristic beginning of a serious disease should arouse among doctors “oncological vigilance.” In the literature there are few cases of patients who searched for help when CNS metastases were stated. Salvatierra et al. showed that at the time of diagnosis of lung cancer metastases in the brain were found in approximately 10% of patients; the next 15–20% of metastatic changes arose during the course of the disease [3]. Symptoms of brain metastases included headache, nausea, vomiting, change in behavior, general weakness and malaise, paralysis of the cranial nerves, aphasia, and seizures. Brain metastases influence survival and quality of life and have economic impact [4].

Our observations were similar. Our patients had proper diagnosis after many CNS examinations. We wanted to emphasize that symptoms which suggested neuroinfections masked lung cancer of two different histopathological backgrounds: small cell cancer and the squamous cell carcinoma. No relation of neurological symptoms with a specific histological type of lung cancer may be stated.

## 5. Conclusions

1. Neurological symptoms in the area endemic for tick-borne diseases suggesting neuroinfection require careful differential diagnosis.
2. Neurological symptoms in heavy smokers may be associated with metastases of lung cancer.
